# Tracheal Duplication Cyst Presenting as Chest Pain

**DOI:** 10.7759/cureus.43285

**Published:** 2023-08-10

**Authors:** Shreyas Bellur, Vivek Bhat, Sreekar Balasundaram

**Affiliations:** 1 Department of Cardiothoracic Surgery, St. John’s Medical College Hospital, Bangalore, IND; 2 Department of Medicine and Surgery, St. John's Medical College, Bangalore, IND

**Keywords:** thoracic surgery, chest pain, foregut cyst, congenital, tracheal duplication cyst

## Abstract

Tracheal duplication cysts (TDCs) are congenital malformations that are rarely diagnosed in adulthood. The authors present a case of a 43-year-old female with no known comorbidities with a two-year history of chest and upper abdominal pain. Her previous imaging on an outpatient basis was suggestive of an esophageal duplication cyst, and she was lost to follow-up until the current admission. She gave a past surgical history of video-assisted thoracoscopic surgery for a "cyst" excision, with the relevant details unavailable. On examination, the findings were unremarkable. Repeat imaging was suggestive of an esophageal duplication cyst with no change in dimensions. She underwent a right-sided elective thoracotomy and cyst excision. Intraoperatively, a smooth globular mass was visualized next to the esophagus below the level of the carina. The biopsy revealed a TDC. The patient had an uneventful postoperative period and was asymptomatic on follow-up after three months.

TDCs pose a diagnostic challenge as they can only be diagnosed by imaging and histopathology. However, when the imaging is atypical, histopathology clinches the diagnosis. Complete surgical excision is recommended for symptomatic patients after ruling out malignancy. Recurrence of the lesion must be considered in patients such as ours.

Our case emphasizes the consideration of TDCs in the differential diagnosis and advocates the importance of complete surgical resection to prevent a recurrence.

## Introduction

Tracheal duplication cysts (TDCs) are extremely rare congenital malformations. They are believed to result from abnormal budding of the bronchial tree in early embryogenesis [1].

TDCs usually present in childhood, with stridor or dysphagia, secondary to mass effect. TDCs detected in adulthood are uncommon [2]. We report a case of a 43-year-old woman who presented with atypical symptoms with a diagnosis of a TDC.

## Case presentation

A 43-year-old female with no known comorbidities presented to our center with complaints of chest pain and upper abdominal pain for two years. The pain was mild in severity, located over the front of her chest, did not radiate, and was described as a dull ache. There was no relation to exertion or stress. She did report occasional difficulty swallowing food. There was no progression of the symptoms. She was previously evaluated for abdominal pain on an outpatient basis. Contrast-enhanced computed tomography (CECT) of the chest and abdomen was suggestive of an esophageal duplication cyst. The patient was lost to follow-up until the current admission where she gave a past surgical history of video-assisted thoracoscopic surgery (VATS) for a "cyst" excision, with the relevant details unavailable.

On examination, she was hemodynamically stable. Physical examination revealed healed scars suggestive of previous thoracic surgery on the chest and no other abnormalities. Her cardiac and respiratory examination was unremarkable.

**Figure 1:**
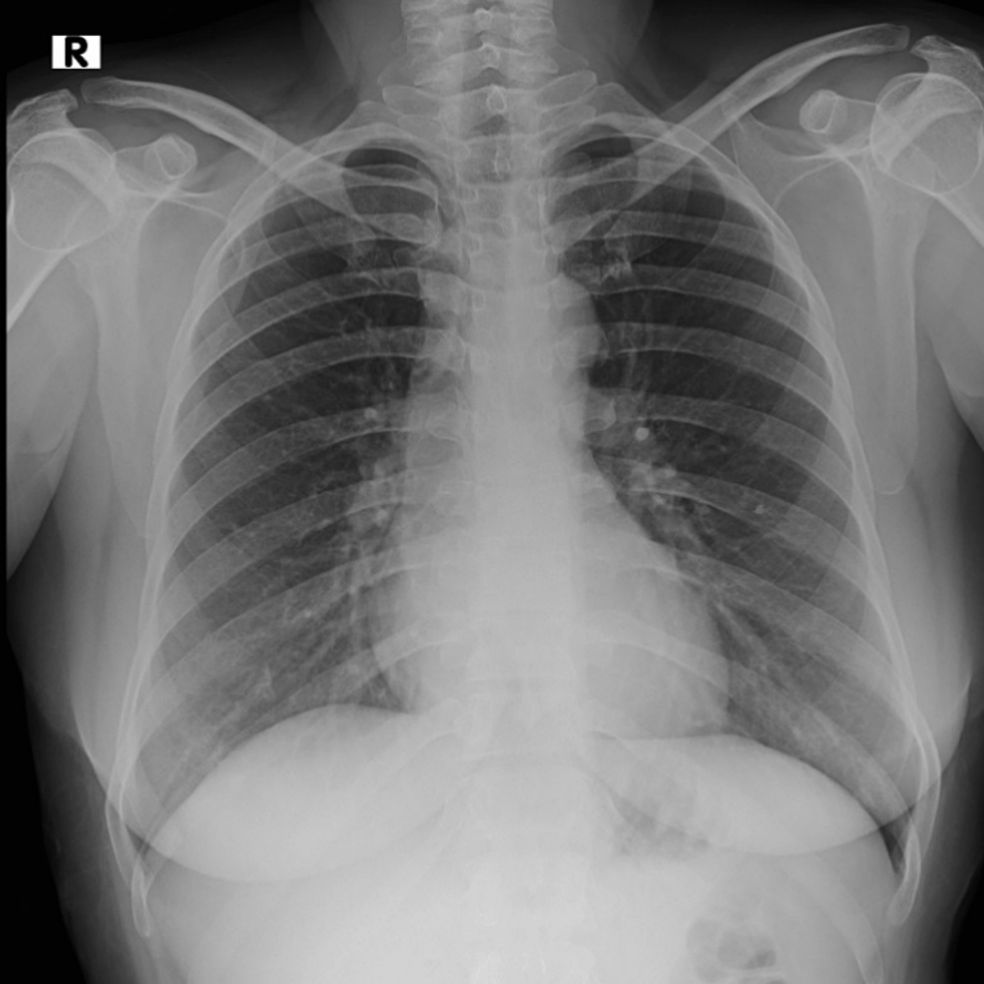
Preoperative chest X-ray showing normal bronchovascular markings

**Figure 2:**
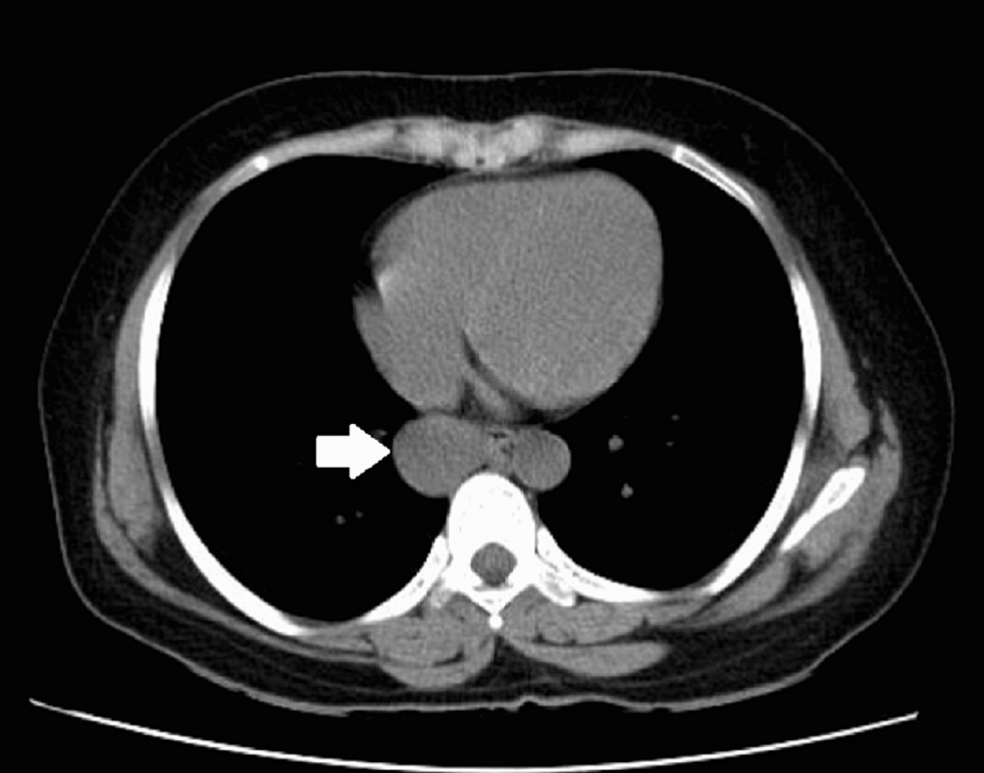
Axial CT of the chest showing a well-defined hypoattenuating lesion in the posterior mediastinum (arrow)

Her biochemical and hematologic laboratory parameters were all within normal limits. Her chest X-ray was normal (Figure 1). A repeat high-resolution computed tomography of the chest revealed a posterior mediastinal lesion that was reported to be likely of esophageal origin (Figure 2). There was no expansion in size or extension, in comparison to the previous reports from outside.

**Figure 3:**
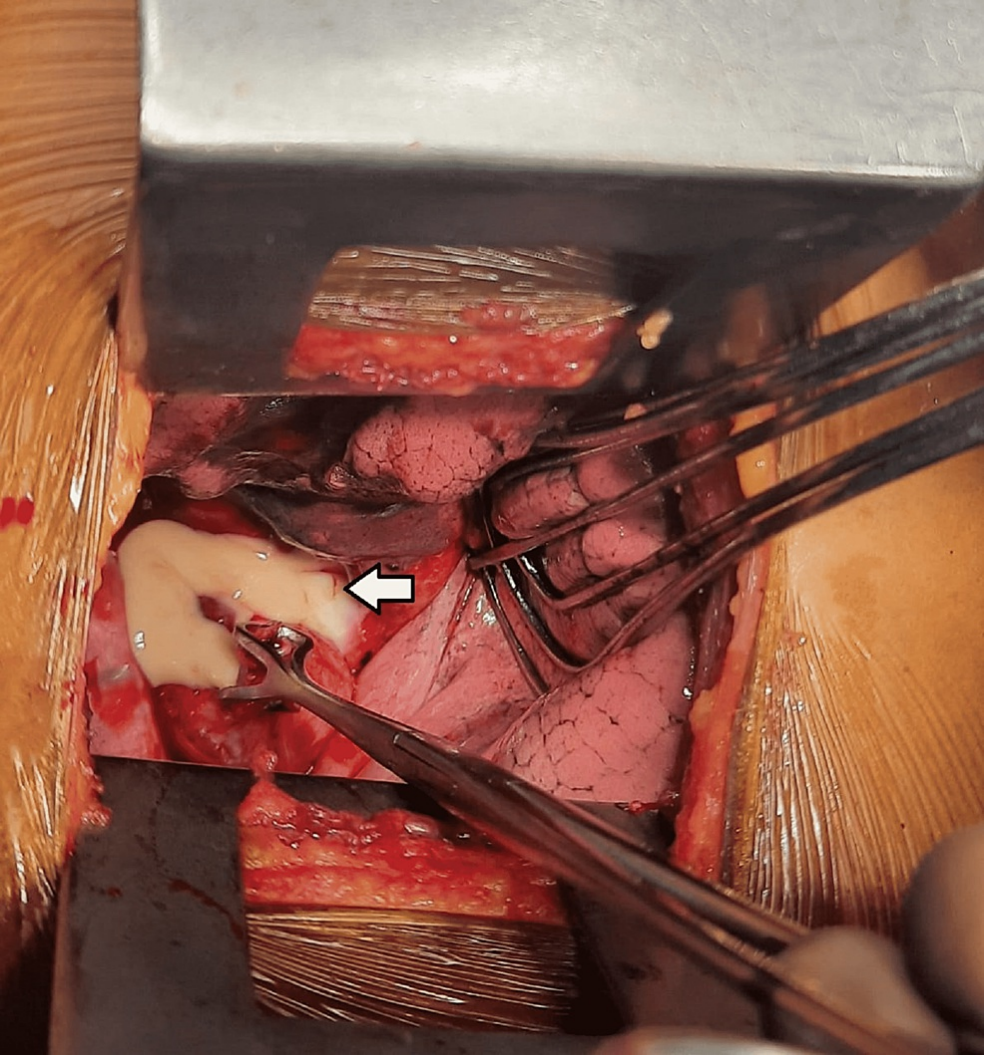
Intraoperative view showing white pultaceous material (arrow) surrounded by the ruptured cyst wall

**Figure 4:**
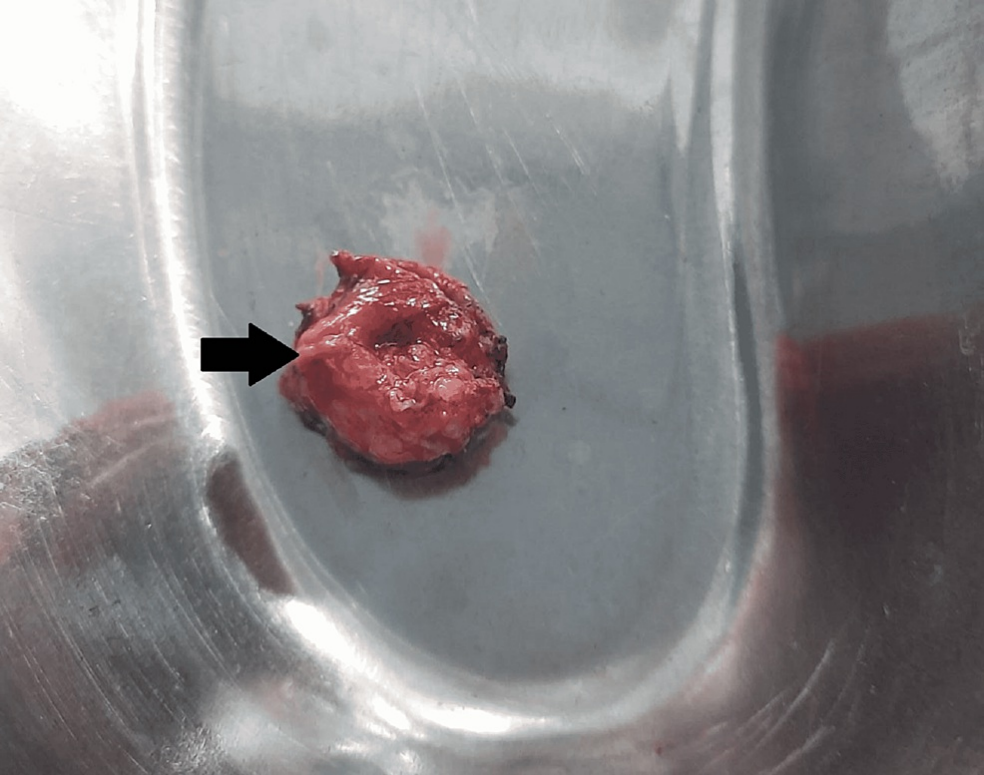
Excised specimen showing the cyst wall (arrow)

She was taken up for elective thoracotomy and excision of the cyst. The approach was from the right side, and intraoperatively, a smooth globular mass measuring roughly 2 x 3 cm was visualized, adjacent to the esophagus well below the level of the carina. However, on dissection, the cyst ruptured and a thick pultaceous material was noted (Figure 3). The cyst was excised in toto and sent for histopathological examination (HPE) along with adjacent lymph nodes (Figure 4). A biopsy of the cyst was taken for HPE.

**Figure 5:**
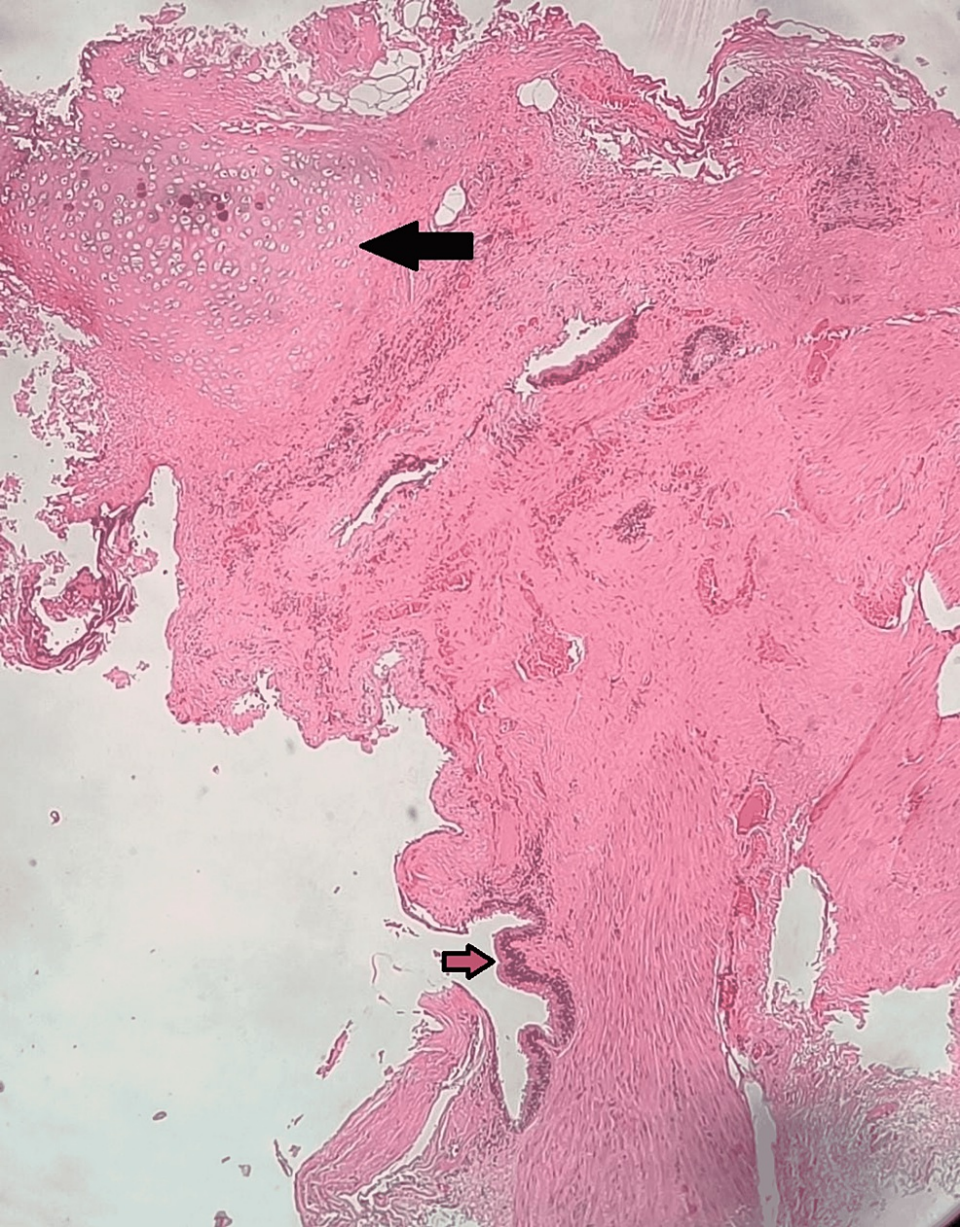
Histopathological specimen showing respiratory epithelium and hyaline cartilage Red arrow: respiratory epithelium; black arrow: hyaline cartilage.

HPE of the cyst wall found fibromuscular tissue lined by pseudostratified columnar epithelium, with underlying subepithelial tissue showing foci of mucinous glands, and portions of cartilage, suggestive of a TDC (Figure 5). The adjacent lymph nodes showed features consistent with reactive hyperplasia.

Her postoperative course was uneventful, and she had no complaints on follow-up.

## Discussion

TDCs diagnosed in adulthood have been reported only a limited number of times in medical literature [1-4].

Symptomatic patients typically present with symptoms due to compression of the trachea and esophagus. Many are asymptomatic. There are no clinical findings specific to TDCs, but congenital anomalies of the vertebra, vasculature, and lung have commonly been reported to coexist [4,5]. Our patient had findings consistent with existing literature. Other than an incidental ovarian follicular cyst and uterine fibroid diagnosed in an earlier evaluation, she had no other anomalies. At presentation, our provisional diagnosis was an esophageal duplication cyst with differentials of paravertebral schwannoma, ganglioneuroma, and bronchogenic cyst considered, given the location of the lesion and the previous history of surgery.

The clinical presentation of TDCs and findings of a well-circumscribed, mediastinal mass on imaging overlap with those of laryngeal duplication cysts and bronchogenic duplication cysts of the trachea [6]. One differentiating factor is the location of the cyst; TDCs are typically located at the level of the tracheal rings whereas laryngeal duplication cysts arise from the thyroid cartilage and bronchogenic cysts from the lung parenchyma with fibrous connection to the lower airway [6]. Our patient’s TDC was located below the level of the carina adjacent to the esophagus and the thoracic vertebra, contributing to diagnostic confusion. Ultimately, HPE is required to clinch the diagnosis. The microscopic findings resemble bronchial tissue, with the classical features of tracheal tissue also present, which include hyaline cartilage, mucinous glands, and the absence of a muscle layer between the mucosa and submucosa [7].

Their mediastinal location makes mediastinal germ cell tumors of particular concern, especially when present in the anterior mediastinum. So, laboratory investigations should ideally include specific tumor markers such as alpha-fetoprotein and beta-human chorionic gonadotropin [8].

The primary approach to TDCs depends on the presence of malignant transformation and metastases [9]. In their absence, complete surgical resection is recommended for symptomatic cases like ours, as chances of recurrence are greatly reduced. The approach is decided based on imaging studies [2]. For our patient, we anticipated adhesions due to her previous surgical history, so we employed a thoracotomy approach. Our patient's history of surgery for "cyst" excision suggests that her presentation to us was possibly a recurrent cyst. However, due to a lack of consistent records, we could not confirm this.

## Conclusions

TDCs are rare congenital malformations that pose a significant diagnostic challenge. TDCs should be considered in the differential diagnosis of posterior mediastinal masses if, on imaging, the location is near the tracheal rings. HPE is necessary to confirm the diagnosis. Due to its rarity, there remains considerable confusion regarding the optimum management of a TDC. Symptomatic cases, such as ours, benefit considerably from complete surgical excision after ruling out malignant transformation.
